# Why have overdose deaths decreased? Widespread fentanyl saturation and decreased drug use among key drivers

**DOI:** 10.1016/j.lana.2025.101226

**Published:** 2025-09-24

**Authors:** Deborah Dowell, Nisha Nataraj, Michaela Rikard, Joohyun Park, Kun Zhang, Grant Baldwin

**Affiliations:** Division of Overdose Prevention, National Center for Injury Prevention and Control, Centers for Disease Control and Prevention, 4700 Buford Highway NE, Atlanta, GA, 30341, United States

**Keywords:** Overdose, Opioids, Fentanyl, Drug use

## Abstract

While overdose deaths remain high in the United States (U.S.), national data show a 25.0% decline in overdose deaths from the year ending in March 2025 compared to the previous year. Reductions since 2015 in the population exposed to overdose risk through drug use may have until recently been offset by an increased per-person mortality risk, driven by replacement of heroin with fentanyl in the drug supply. We estimated overdose deaths and counterfactual scenarios from 2016 to 2023. An estimated 109,783 additional people would have died from opioid overdose if the population exposed to opioid overdose risk had remained constant rather than declining; an estimated 260,024 fewer people would have died from overdose if probability of fentanyl involvement in opioid overdose deaths had remained constant rather than increasing. Fentanyl's representation in the U.S. drug supply appears to be a key driver of overdose trends. A declining population exposed to overdose risk over the last decade may be related to prior deaths and to evidence-based efforts to prevent substance use and opioid use disorder.

## Introduction

**Fig. 1:**
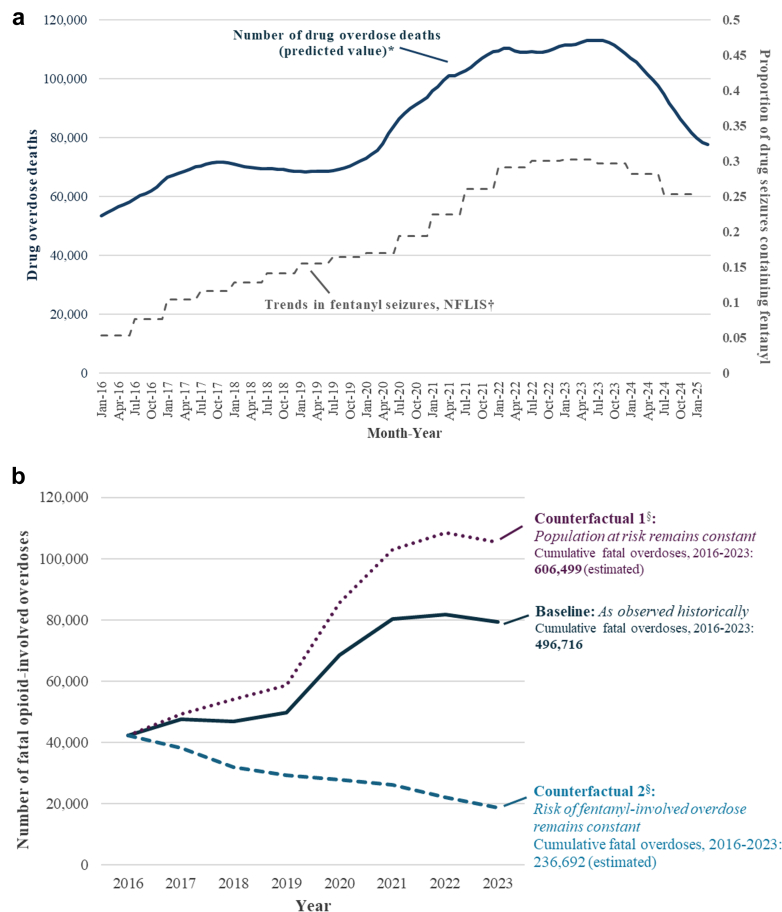
**a.** Historical trends in overdose deaths and fentanyl seizures. **b.** Estimated counterfactual outcomes. **Abbreviations:** NFLIS, National Forensic Laboratory Information System. ∗Drug overdose deaths are shown for the 12-month period ending at each month-year on the x-axis. Data^1^ are provisional and may be subject to minor revision when finalized. ^†^Data from.^2^ Data for second half of 2024 may represent undercounts given proportions may change with reporting of final counts. ^§^Data on counterfactual scenarios are available in the Appendix and were obtained by applying an extended heuristic based on.^3^

United States national data show persistent declines in overdose deaths beginning in 2023 (Fig. 1 Panel a). Provisional data indicate a 25.0% decline in overdose deaths from the year ending in March 2025 (77,648 overdose deaths) versus the previous year (103,529),^1^ a dramatic shift from relentlessly rising overdose deaths for much of the past 25 years. Most overdose deaths involve opioids (66.2% in the year ending in March 2025); 57.9% involve synthetic opioids including fentanyl (including fentanyl analogs).^1^ Opioid overdose deaths remain alarmingly high (more than 140 per day for the year ending in March 2025). Still, there is reason for optimism.

## Widespread fentanyl saturation and decreased drug use among key drivers

What explains the recent substantial drop in overdose deaths in the U.S.? Two crucial drivers may be less widespread drug use and changes in the fentanyl supply. With U.S. prescription opioid sales declining by more than a third from 2011 to 2019^4^ and further declines from a rate of 46.8 opioid prescriptions dispensed per 100 persons in 2019 to 37.5 opioid prescriptions dispensed per 100 persons in 2023,^5^ fewer people have been exposed to prescription opioids in recent years, potentially curbing the development of opioid use disorder (OUD), opioid misuse, and use of opioids other than as prescribed.^4^ While consistent data on numbers of people at risk of overdose in the U.S. over time are limited, National Survey on Drug Use and Health (NSDUH) data show estimated prevalence of OUD (including prescription OUD and heroin use disorder) decreased from 2.1 million in 2016 to 1.6 million in 2019^6^ and show reductions in people among people aged ≥12 years misusing prescription opioids or using illegal opioids from 2015 to 2023.^7^, ^8^, ^9^ Models published in 2021^4^ and 2022^10^ predicted a decline in overdose deaths due to reduced OUD onset^4^ and increased awareness of opioid risks.^10^ One model predicted deaths involving opioids would decline after peaking in 2023,^10^ similar to observed trends (Fig. 1 Panel a). The population reporting drug use might also decline through treatment with medications for OUD, which are effective in reducing illegal opioid use.^11^ However, use of these medications remains relatively infrequent (about 25% of the population who could benefit from them).^12^ Tragically, the population reporting drug use might also have declined through prior deaths from overdose as the individuals at greatest risk for overdose were increasingly exposed to fentanyl in recent years.^13^

Any reduction in the number of individuals exposed to overdose risk (population at risk) through drug use may have been offset by an increased per-person mortality risk, driven by changes in the drug supply during this period. Starting in 2013, fentanyl began replacing heroin in the Eastern U.S. and spread Westward, driving profound increases in overdose deaths,^14^ with the rate of increase accelerating during 2020 and 2021,^1^ coincident with, and likely related to, the COVID-19 pandemic emergency.^15^

A concept that may account for both the rapid escalation and the more recent decline in overdose rates over the past decade is fentanyl saturation—with fentanyl largely replacing other illegal opioids in the drug market. Once fentanyl has nearly completely replaced heroin (or “saturated” the illegal opioid supply), there are much more limited opportunities for new introduction of fentanyl to people who are already using opioids. Each use of illegal opioids continues to carry a high risk of death, but the risk may be no longer increasing over time since there are fewer transitions to fentanyl from less potent opioids. If fentanyl's share of the illegal drug supply decreases or remains stable within the context of a reduced number of individuals exposed to opioids, this could translate to reduced overdoses and deaths. National Forensic Laboratory Information System (NFLIS) data show a plateauing proportion of law enforcement drug seizures that contained fentanyl by 2023 (Fig. 1 Panel A),^2^^,^^16^ suggesting that fentanyl's share of the drug supply was no longer increasing. Overdose deaths have declined most in Eastern states and are still increasing in some Western states^1^ where fentanyl was introduced later and may not yet have saturated the illegal drug supply.

Additional influences likely contribute, such as naloxone distribution among laypersons,^17^ adulteration of the fentanyl supply with less lethal substances (for example, with xylazine),^18^ more people avoiding fentanyl injection through other, possibly less lethal,^19^ routes like smoking,^20^ and easing of pandemic-related disruptions (to trusted drug suppliers, treatment availability, and use of drugs with people instead of alone).^15^

## Exploration of key drivers and counterfactual scenarios

To explore potential explanations for changes in opioid overdose deaths between 2016 and 2023, we used a previously published^3^ simple heuristic (Appendix) to estimate overdose deaths as a product of three factors: population at risk of opioid overdose, number of overdoses per person at risk, and probability of an overdose being fatal. Each of these factors may be modified through evidence-based public health interventions or other changes. For example, increased drug supply potency (e.g., replacement of heroin with fentanyl) increases both number of overdoses and proportion of overdoses that are fatal, while prevention efforts can reduce the number of people who use drugs and naloxone can reduce the chances that an overdose is fatal. We extended this heuristic to separately consider fentanyl-involved overdose and examined simple counterfactual scenarios estimating fatal opioid overdoses assuming no changes since 2016 in population exposed to overdose risk and probability of fentanyl-involved overdose. For the population exposed, we used estimates from NSDUH of people aged ≥12 years misusing prescription opioids or using illegal opioids in the past year.^7^, ^8^, ^9^ Fatal overdose data from the National Vital Statistics System^21^ and nonfatal overdose data from the Centers for Disease Control and Prevention (CDC) Drug Overdose Surveillance and Epidemiology (DOSE) System^22^ and scientific literature^23^ were used to estimate overdoses per person at risk and probability of fatal overdose.

Counterfactual scenarios where numbers of people at risk of opioid overdose remained constant from 2016 to 2023 estimated that 109,783 additional people (606,499 instead of the 496,716 deaths observed) would have died from opioid overdose (Fig. 1 Panel b). The declining population exposed to overdose risk over the last decade may be related to prevention efforts, including implementation of evidence-based strategies to prevent or reduce substance use. Campaigns like CDC's Stop Overdose and the Drug Enforcement Administration's One Pill Can Kill complement widespread efforts by health departments, community-based organizations, schools, and others to raise awareness about the dangers of fentanyl. The 2022 CDC Clinical Practice Guideline for Prescribing Opioids for Pain^24^ and other health system efforts have helped prevent OUD by promoting safer pain management, reducing unnecessary opioid use, and encouraging nonopioid alternatives.

Counterfactual scenarios where probability of fentanyl-involved overdose remained constant from 2016 to 2023 estimated that 260,024 fewer people (236,692 instead of the 496,716 deaths observed) would have died from overdose (Fig. 1 Panel b). Fentanyl's proliferation in the U.S. drug supply appears to be a key driver of overdose trends, increasing the risk of overdose and death for those exposed to opioids. It is unclear whether fentanyl's recent plateau and decline are due to saturation or other factors, such as replacement of fentanyl with xylazine and other drugs, possibly indicating reduced fentanyl or precursor availability in the supply chain.

Scenario limitations include year-to-year variations in data sources, likely underestimates for the population at risk for opioid overdose, including people exposed to fentanyl through use of non-opioid drugs, variable lag periods between opioid exposure and overdose risk,^4^ incomplete national coverage for overdose trends, and the assumption that the heuristic factors are independent, which might not remain true over the longer term. Increased awareness, testing, and introduction of a specific code for fentanyl (replacing the broader code for synthetic opioids in October 2020)^25^ might have increased reported fentanyl-involved overdoses. Still, the pattern of increasing fentanyl involvement followed by a plateau in 2023 aligns with fentanyl representation in drug seizure data (Fig. 1 Panel A) and among overdoses in a subset of patients tested for fentanyl (A. Manini, personal communication regarding Fentalog study^26^ data, May 5, 2025). We did not include counterfactual outcomes beyond 2023 given more recent data on population at risk for opioid overdose were not available. Finally, NFLIS data (Fig. 1 Panel a) from the last half of 2024 may be incomplete.

## Conclusions

Our counterfactual scenarios highlight how changes in the drug supply can lead to substantial changes in overdose deaths and at the same time, how reductions in the numbers of people using drugs can translate to fewer overdoses than would have occurred had this population remained constant. Overdose rates remain alarmingly high, and a sustained downward trend is not guaranteed. Efforts to limit access to precursor chemicals for illegal fentanyl production and strengthening law enforcement actions targeting counterfeit pill manufacturing and distribution might further recent reductions in lethality of the drug supply.^27^ At the same time, any future increases in the fentanyl concentration in the drug supply, or continued introduction of even more potent substances, including fentanyl analogs such as carfentanil, have the potential to increase overdose deaths.^28^ The unpredictability of changes in the illegal drug supply highlights the importance of continued vigilance and of a comprehensive multisectoral approach to prevent drug use and its harmful effects. To enable earlier and more actionable identification of emerging trends, CDC's Overdose Data to Action^29^ supports state, territorial, county, and city health departments in expanding access to real-time, localized data. Continued implementation of evidence-based programs and policies, including wider naloxone distribution to prevent fatal overdoses among the large population still exposed to fentanyl and other opioids^30^ as well as education, appropriate pain management,^24^ and increased access to medications for OUD, can reduce drug use^11^ and use disorders^4^ to limit the population exposed to potentially lethal illegal drugs.

## Contributors

DD: conceptualisation, data curation, supervision, validation, writing—original draft, and writing—review & editing; NN: conceptualisation, data curation, formal analysis, methodology, validation, visualisation, writing—original draft, and writing– review & editing; MR: conceptualisation, data curation, formal analysis, methodology, validation, visualisation, and writing—review & editing; JP: data curation, formal analysis, methodology, validation, visualisation, and writing—review & editing; KZ: conceptualisation, data curation, methodology, supervision, validation, and writing—review & editing; GB: conceptualisation, writing—original draft, and writing—review & editing.

DD, NN, MR, JP, and KZ have accessed and verified the data. DD, NN, MR, JP, KZ, and GB were responsible for the decision to submit the manuscript.

## AI use statement

Artificial intelligence tools were not used in writing or analysis for this manuscript.

## Declaration of interests

The authors declare no competing interests.

The views expressed in this article are those of the authors and do not necessarily represent the official position of the Centers for Disease Control and Prevention.
