# Supplementary material for: Why have overdose deaths decreased? Widespread fentanyl saturation and decreased drug use among key drivers
Source: Lancet Reg Health Am. 2025 Sep 24;51:101226. doi: 10.1016/j.lana.2025.101226 (PMC12664646; doi:10.1016/j.lana.2025.101226)
Supplement: Appendix [file mmc1.docx]

**Appendix: Supplementary material**

**Why have overdose deaths decreased?**

**Widespread fentanyl saturation and decreased drug use among key drivers**

Deborah Dowell, Nisha Nataraj, Michaela Rikard, Joohyun Park, Kun Zhang, and Grant Baldwin

**A. Extended heuristic**

We were interested in exploring plausible reasons for the changes in fatal opioid overdoses between 2016-2023 and possibly beyond, including changing share of fentanyl in the illegal drug supply and population using opioids (i.e., population at risk for opioid overdose). Skinner et al. (2024)^1^ developed a heuristic to estimate the number of fatal overdoses (D) as a function of three terms, the exposed population at risk of opioid overdose (N), the number of opioid overdoses per person at risk (β), and the probability of an opioid overdose being fatal (μ), i.e., *D = Nβμ*. As the authors emphasize, each of these factors can be addressed through public health interventions or other changes. We extended this heuristic to consider fentanyl-involved overdoses and utilized simple counterfactual analyses to estimate fatal overdoses if there were no change in either 1) the population at risk, or 2) fentanyl-involved opioid overdoses (fatal and nonfatal) between 2016-2023.

To extend the heuristic, we first expressed opioid-involved overdoses as the sum of number of fatal ($O_{fatal})$ and nonfatal ($O_{nonfatal})$ opioid-involved overdoses impacting the terms β and μ (Eq. 2). Next, we separately considered fatal overdoses involving fentanyl ${(O}_{fatal, fent})$ or other opioids ($O_{fatal, \bar{fent}})$ and nonfatal overdoses involving fentanyl ($O_{nonfatal, fent}$) or other opioids ($O_{nonfatal,\bar{fent}}$) (Eq. 3 and 4). The final extended heuristic distinguishes between fentanyl- ($\boldsymbol{\beta}_{\boldsymbol{fent}}\boldsymbol{)}$vs. other opioid-involved $\boldsymbol{(\beta}_{\bar{\boldsymbol{fent}}}\boldsymbol{)}$ overdoses per person at risk, and probability of fentanyl- ($\boldsymbol{\mu}_{\boldsymbol{fent}}\boldsymbol{)}$vs. other opioid-involved $\boldsymbol{(\mu}_{\bar{\boldsymbol{fent}}}\boldsymbol{)}$ overdose (Eq. 5).

$\boldsymbol{D=N\beta\mu}$ (Eq. 1)

$=N\left[ \frac{O_{fatal} +O_{nonfatal}}{N} \right]\left[ \frac{O_{fatal}}{O_{fatal} +O_{nonfatal}} \right]$ (Eq. 2)

$=N\left[ \frac{O_{fatal, fent} +O_{fatal, \bar{fent}} +O_{nonfatal, fent}+ O_{nonfatal,\bar{fent}}}{N} \right]$ $\left[ \frac{O_{fatal, fent}+O_{fatal, \bar{fent}}}{O_{fatal, fent} +O_{fatal, \bar{fent}} +O_{nonfatal, fent}+ O_{nonfatal,\bar{fent}}} \right]$ (Eq. 3)

$=N\left[ \frac{{(O}_{fatal, fent} +{O_{nonfatal, fent}) +(O}_{fatal, \bar{fent}} + O_{nonfatal, \bar{fent}})}{N} \right]*\left[ \frac{O_{fatal, fent}+O_{fatal, \bar{fent}}}{O_{fatal, fent} +O_{fatal, \bar{fent}} +O_{nonfatal, fent}+ O_{nonfatal, \bar{fent}}} \right]$ (Eq. 4)

$\boldsymbol{D =N}{\boldsymbol{(}\boldsymbol{\beta}_{\boldsymbol{fent}}\boldsymbol{+\beta}}_{\bar{\boldsymbol{fent}}}\boldsymbol{)}{\boldsymbol{(}\boldsymbol{\mu}_{\boldsymbol{fent}}\boldsymbol{+\mu}}_{\bar{\boldsymbol{fent}}}\boldsymbol{)}$ (Eq. 5)

**B. Estimating nonfatal overdoses nationally**

We obtained national estimates of nonfatal overdoses involving fentanyl and other opioids from 2016 to 2020 using a previous study^2^ that analyzed emergency department (ED) data from the Healthcare Cost and Utilization Project Nationwide Emergency Department Sample (NEDS). Fentanyl-involved nonfatal overdoses were defined as nonfatal drug overdoses involving synthetic opioids, identified using ICD-10-CM codes (T40.4X, T40.41, T40.42, T40.49), as fentanyl-specific ICD-10-CM codes were not available until October 2020. Nonfatal overdoses involving other opioids were calculated by subtracting synthetic opioids-related overdoses from the total opioids-related overdoses (ICD-10-CM codes, T40.0X, T40.1X, T40.2X, T40.3X, T40.4X, T40.41, T40.42, T40.49, T40.60, T40.69) using the data reported in Casillas et al.^2^

To estimate nonfatal overdoses between 2021-2023, we additionally used ED discharge data from CDC’s Drug Overdose Surveillance and Epidemiology (DOSE) system.^3^ The DOSE discharge data represents ED visits for nonfatal overdoses involving fentanyl and all opioids, using the same ICD-10-CM codes as those used in Casillas et al.,^2^ from the 27 jurisdictions (AK, AZ, CA, CO, DC, FL ,GA, HI, IA, IL, IN, KY, LA, MD, MI, MN, MO, MS, MT, NC, NE, NJ, OR, RI, SC, SD, WI) that consistently reported ED data from 2018 through 2023. The three jurisdictions that did not report continuous data for all six years were excluded from this analysis. We first used the DOSE ED discharge data for the years 2018-2023. For the years 2018-2020, we calculated the proportion of all opioid-involved nonfatal overdoses from the DOSE discharge data relative to the national overdose data from HCUP data, as reported in Casillas et al.^2^ We then averaged these proportions across 2018-2020 to estimate the proportion of national nonfatal overdoses captured in the DOSE system each year between 2021-2023 (estimated proportion: 0.55, which is similar to the proportion of states included in the DOSE discharge data versus all 50 states and the District of Columbia, 27/51=0.53). We repeated the same analysis for fentanyl-involved nonfatal overdoses between 2018-2020 (estimated proportion 0.54). We applied these proportions (0.55 for all opioids and 0.54 for fentanyl) to estimate national nonfatal overdose involving all opioids and fentanyl for the year 2021-2023. Finally, we estimated the number of other opioid-involved nonfatal overdoses by calculating the difference between the estimated nonfatal overdoses involving fentanyl and all opioids, as we did for the years 2018-2020 (eTable 1).

eTable 1 - Estimating national nonfatal overdoses using Emergency Department discharge data from CDC’s Drug Overdose Surveillance and Epidemiology (DOSE) system^3^ and published estimates,^2^ 2021-2023

| **Year** | **DOSE: All nonfatal opioid overdoses*** | **DOSE: Fentanyl-involved nonfatal overdoses*** | **DOSE: Other opioid-involved nonfatal overdoses*** | **Estimated US opioid-involved nonfatal overdoses†** | **Estimated US fentanyl-involved nonfatal overdoses†** | **Estimated US other-opioid involved nonfatal overdoses††** |
| --- | --- | --- | --- | --- | --- | --- |
| 2021 | 183,245 | 27,648 | 155,597 | 331,788 | 51,210 | 280,578 |
| 2022 | 169,197 | 34,505 | 134,692 | 306,352 | 62,476 | 243,877 |
| 2023 | 157,002 | 41,212 | 115,790 | 284,272 | 74,619 | 209,652 |
| * Annual counts of nonfatal overdose-involved emergency department visits, by drug type, 2021-2023 CDC DOSE discharge data, 27 jurisdictions (AK, AZ, CA, CO, DC, FL ,GA, HI, IA, IL, IN, KY, LA, MD, MI, MN, MO, MS, MT, NC, NE, NJ, OR, RI, SC, SD, WI). Fentanyl-involved nonfatal overdoses were defined as nonfatal drug overdoses involving synthetic opioids, identified using ICD-10-CM codes (T40.4X, T40.41, T40.42, T40.49). Nonfatal overdoses involving other opioids were calculated by subtracting synthetic opioids-related overdoses from the total opioids-related overdoses (ICD-10-CM codes, T40.0X, T40.1X, T40.2X, T40.3X, T40.4X, T40.41, T40.42, T40.49, T40.60, T40.69) | | | | | | |
| † Estimated US nonfatal opioid- and fentanyl-involved nonfatal overdoses calculated based on the estimated proportion of nonfatal overdoses represented in the CDC’s DOSE Emergency Department discharge data^3^ relative to the national overdose data from the Nationwide Emergency Department Sample (NEDS), as reported in Casillas et al.^2^ | | | | | | |
| †† Estimated US nonfatal other-opioid involved overdoses estimated from the difference between the estimated nonfatal overdoses involving fentanyl and all opioids. | | | | | | |

**C. Fatal and nonfatal overdoses over time**

eTable 2 - Fatal and nonfatal overdoses involving fentanyl and other opioids, 2016-2023

| **Year** | **Fatal overdoses, any opioid* (D)** | **Fatal overdoses, fentanyl involved***  $\boldsymbol{(O}_{\boldsymbol{fatal, fent}}\boldsymbol{)}$ | **Fatal overdoses, other opioid involved***  **(**$\boldsymbol{O}_{\boldsymbol{fatal,}\bar{\boldsymbol{fent}}}\boldsymbol{)}$ | **Nonfatal overdoses, any opioid**** | **Nonfatal overdoses, fentanyl involved****  $\boldsymbol{(O}_{\boldsymbol{nonfatal, fent}}$**)** | **Nonfatal overdoses, other opioid-involved****  **(**$\boldsymbol{O}_{\boldsymbol{nonfatal,}\bar{\boldsymbol{fent}}}$**)** |
| --- | --- | --- | --- | --- | --- | --- |
| 2016 | 42,249 | 19,413 | 22,836 | 289,685 | 17,610 | 272,075 |
| 2017 | 47,600 | 28,466 | 19,134 | 301,643 | 19,506 | 282,137 |
| 2018 | 46,802 | 31,335 | 15,467 | 270,873 | 20,221 | 250,652 |
| 2019 | 49,860 | 36,359 | 13,501 | 269,986 | 23,350 | 246,636 |
| 2020 | 68,630 | 56,516 | 12,114 | 295,649 | 33,670 | 261,979 |
| 2021 | 80,411 | 70,601 | 9,810 | 331,788 | 51,210 | 280,578 |
| 2022 | 81,806 | 73,838 | 7,968 | 306,352 | 62,476 | 243,877 |
| 2023 | 79,358 | 72,776 | 6,582 | 284,272 | 74,619 | 209,652 |
| * National Center for Health Statistics, National Vital Statistics System, mortality data file.^4^ Among deaths with drug overdose as the underlying cause, the following multiple cause-of-death codes indicate the drug type(s) involved: Any opioid (T40.0–T40.4, T40.6), fentanyl-involved: synthetic opioids other than methadone (T40.4), other opioid-involved (T40.0–T40.3, T40.6) | | | | | | |
| **Nonfatal overdoses from 2016-2020 obtained from published estimates^3^ and 2021-2023 obtained from adjusted CDC’s DOSE ED discharge data^3^ (see B.) | | | | | | |

**D. Population at risk and number and probability of fentanyl-involved and other opioid-involved overdoses**

eTable 3 - Estimated heuristic inputs - population at risk for opioid overdose*, number of fentanyl-involved and other opioid overdoses per person at risk**, probability of fentanyl-involved and other opioid involved fatal overdose***, 2016-2023

| **Year** | **Population at risk, in 1000s (N)*** | **Fentanyl-involved overdoses per person at risk (**$\boldsymbol{\beta}_{\boldsymbol{fent}}\boldsymbol{)}$ | **Other opioid-involved overdoses per person at risk** $\boldsymbol{(\beta}_{\bar{\boldsymbol{fent}}}\boldsymbol{)}$ | **Probability of fentanyl-involved fatal overdose**  **(**$\boldsymbol{\mu}_{\boldsymbol{fent}}\boldsymbol{)}$ | **Probability of other opioid-involved fatal overdose**  $\boldsymbol{(\mu}_{\bar{\boldsymbol{fent}}}\boldsymbol{)}$ |
| --- | --- | --- | --- | --- | --- |
| 2016 | 11,824 | 0·0031 | 0·0249 | 0·0585 | 0·0688 |
| 2017 | 11,401 | 0·0042 | 0·0264 | 0·0815 | 0·0548 |
| 2018 | 10,250 | 0·0050 | 0·0260 | 0·0986 | 0·0487 |
| 2019 | 10,065 | 0·0059 | 0·0258 | 0·1137 | 0·0422 |
| 2020 | 9,490 | 0·0095 | 0·0289 | 0·1551 | 0·0333 |
| 2021 | 9,236 | 0·0132 | 0·0314 | 0·1713 | 0·0238 |
| 2022 | 8,918 | 0·0153 | 0·0282 | 0·1902 | 0·0205 |
| 2023 | 8,902 | 0·0166 | 0·0243 | 0·2001 | 0·0181 |
| *Estimates of people aged ≥12 years misusing prescription opioids or using illegal opioids in the past year obtained from National Survey on Drug Use and Health Detailed Tables:  [1] Table 7.2A – Types of Illicit Drug, Tobacco Product, and Alcohol Use in Past Year: Among People Aged 12 or Older; Numbers in Thousands, 2002-2020. Available from <https://www.samhsa.gov/data/report/2020-nsduh-detailed-tables> (data for 2015-2020)  [2] Table 1.1A – Types of Illicit Drug Use in Lifetime, Past Year, and Past Month: Among People Aged 12 or Older. Available from <https://www.samhsa.gov/data/report/2021-nsduh-detailed-tables> (data for 2021)  [3] Table 1.1A – Types of Illicit Drug Use in Lifetime, Past Year, and Past Month: Among People Aged 12 or Older; Numbers in Thousands**,** 2022 and 2023. Available from <https://www.samhsa.gov/data/sites/default/files/reports/rpt47100/NSDUHDetailedTabs2023_v1/NSDUHDetailedTabs2023_v1/2023-nsduh-detailed-tables-sect1pe.htm#tab1.1a> (data for 2022, 2023)  ** Number of opioid overdoses per person at risk is expressed as the total number of fatal and estimated nonfatal overdoses divided by the number of people at risk  *** Probability of a fatal overdose is the number of fatal overdoses divided by the total number of fatal and estimated nonfatal overdoses | | | | | |

**E. Counterfactual scenarios using extended heuristic**

eTable 4 - Counterfactual outcomes between 2016-2023 estimated through extended heuristic

|  |  |  | **Counterfactual 1:** Constant population at risk, 2016-2023 | | **Counterfactual 2:** Constant fentanyl involved fatal overdoses, 2016-2023 | |
| --- | --- | --- | --- | --- | --- | --- |
| **Year** | **Fatal overdoses, total* (**$\boldsymbol{D}$**)** | **Annual change in fatal overdose deaths (%)** | **Est. fatal overdoses (**$\boldsymbol{D}_{\boldsymbol{c}\boldsymbol{1}}$**)** | **Est. annual change in fatal overdoses (%)** | **Est. fatal overdoses (**$\boldsymbol{D}_{\boldsymbol{c}\boldsymbol{2}}$**)** | **Est. annual change in fatal overdoses (%)** |
| 2016 | 42,249 |  | 42,249 |  | 42,249 |  |
| 2017 | 47,600 | 12·7% | 49,366 | 16·8% | 38,169 | -9·7% |
| 2018 | 46,802 | -1·7% | 53,989 | 9·4% | 31,960 | -16·3% |
| 2019 | 49,860 | 6·5% | 58,574 | 8·5% | 29,368 | -8·1% |
| 2020 | 68,630 | 37·6% | 85,509 | 46·0% | 27,871 | -5·1% |
| 2021 | 80,411 | 17·2% | 102,943 | 20·4% | 26,274 | -5·7% |
| 2022 | 81,806 | 1·7% | 108,463 | 5·4% | 22,105 | -15·9% |
| 2023 | 79,358 | -3·0% | 105,407 | -2·8% | 18,695 | -15·4% |
| Overall | 496,716 |  | 606,499 |  | 236,692 |  |
| Change in deaths: counterfactual est. relative to actual N, (%) |  |  |  | 109,783 (22.1%) |  | -260,024  (-52·3%) |
| Abbreviations: Estimated (Est.) | | | | | | |
| * National Center for Health Statistics, National Vital Statistics System, mortality data file.^4^ Among deaths with drug overdose as the underlying cause, the following multiple cause-of-death codes indicate the drug type(s) involved: Any opioid (T40.0–T40.4, T40.6) | | | | | | |

References

1. Skinner A, Nolen S, Cerdá M, Rich JD, Marshall BDL. A simple heuristic for allocating opioid settlement funding to reduce overdose mortality in the United States. Am J Drug Alcohol Abuse*.* 2024;50(3):269-275.
2. Casillas SM, Pickens CM, Tanz LJ, Vivolo-Kantor AM. Estimating the ratio of fatal to non-fatal overdoses involving all drugs, all opioids, synthetic opioids, heroin or stimulants, USA, 2010-2020. Inj Prev 2024;30(2):114-124.
3. Centers for Disease Control and Prevention. Drug Overdose Surveillance and Epidemiology (DOSE) System: Nonfatal Overdose Emergency Department Discharge Data [Internet]. Available from: https://www.cdc.gov/overdose-prevention/data-research/facts-stats/dose-dashboard-nonfatal-discharge-data.html. Accessed April 23, 2025

Available from: <https://www.cdc.gov/overdose-prevention/data-research/facts-stats/dose-dashboard-nonfatal-surveillance-data.html>.

Garnett MF, Miniño AM. Drug overdose deaths in the United States, 2003–2023. NCHS Data Brief, no 522 [Internet]. Hyattsville, MD: National Center for Health Statistics. 2024 Dec 19 (cited 2025 Apr 23). Available from: <https://dx.doi.org/10.15620/cdc/170565>.
